# Measuring Melanoma Nanomechanical Properties in Relation to Metastatic Ability and Anti-Cancer Drug Treatment Using Scanning Ion Conductance Microscopy

**DOI:** 10.3390/cells12192401

**Published:** 2023-10-04

**Authors:** Emily Woodcock, Peter V. Gorelkin, Philip S. Goff, Christopher R. W. Edwards, Yanjun Zhang, Yuri Korchev, Elena V. Sviderskaya

**Affiliations:** 1 Molecular and Clinical Sciences Research Institute, St George’s, University of London, London SW17 0RE, UK; ewoodcoc@sgul.ac.uk (E.W.);; 2 Department of Medicine, Imperial College London, W12 0NN London, UKy.korchev@imperial.ac.uk (Y.K.); 3 Research Laboratory of Biophysics, National University of Science and Technology MISiS, Moscow 119049, Russia; 4 Nano Life Science Institute (WPI-Nano LSI), Kanazawa University, Kakuma-machi, Kanazawa 920-1192, Japan

**Keywords:** melanoma, cell stiffness, SICM, cellular mechanics, cancer, nanomechanics

## Abstract

A cell’s mechanical properties have been linked to cancer development, motility and metastasis and are therefore an attractive target as a universal, reliable cancer marker. For example, it has been widely published that cancer cells show a lower Young’s modulus than their non-cancerous counterparts. Furthermore, the effect of anti-cancer drugs on cellular mechanics may offer a new insight into secondary mechanisms of action and drug efficiency. Scanning ion conductance microscopy (SICM) offers a nanoscale resolution, non-contact method of nanomechanical data acquisition. In this study, we used SICM to measure the nanomechanical properties of melanoma cell lines from different stages with increasing metastatic ability. Young’s modulus changes following treatment with the anti-cancer drugs paclitaxel, cisplatin and dacarbazine were also measured, offering a novel perspective through the use of continuous scan mode SICM. We found that Young’s modulus was inversely correlated to metastatic ability in melanoma cell lines from radial growth, vertical growth and metastatic phases. However, Young’s modulus was found to be highly variable between cells and cell lines. For example, the highly metastatic cell line A375M was found to have a significantly higher Young’s modulus, and this was attributed to a higher level of F-actin. Furthermore, our data following nanomechanical changes after 24 hour anti-cancer drug treatment showed that paclitaxel and cisplatin treatment significantly increased Young’s modulus, attributed to an increase in microtubules. Treatment with dacarbazine saw a decrease in Young’s modulus with a significantly lower F-actin corrected total cell fluorescence. Our data offer a new perspective on nanomechanical changes following drug treatment, which may be an overlooked effect. This work also highlights variations in cell nanomechanical properties between previous studies, cancer cell lines and cancer types and questions the usefulness of using nanomechanics as a diagnostic or prognostic tool.

## 1. Introduction

Scanning ion conductance microscopy (SICM) is a technique capable of imaging live cells with nanoscale resolution whilst simultaneously measuring a range of biological traits, such as pH [1] and ROS [2]. SICM employs a glass nanopipette as a probe to scan the surface of living cells and has also been adapted as a powerful tool for measuring the mechanical properties of live cells under physiological conditions [3,4,5]. The mechanical properties of cells are presented as Young’s modulus (pascals) with a higher Young’s modulus representing a stiffer cell. More recently, an alternative method for nanomechanical data acquisition using SICM has been described using a decane droplet to estimate the intrinsic force between the nanopipette tip and sample surface [6].

SICM for nanomechanical measurements offers advantages over the widely used atomic force microscopy (AFM) due to its non-contact, high-resolution image acquisition and nanomechanical mapping. This is of particular importance when performing repetitive scans to investigate Young’s modulus changes over time. A comprehensive comparison of SICM and AFM for live cell imaging has been previously published by Seifert and colleagues [7]. Furthermore, the value of indentation for SICM nanomechanical measurements can be of an order of magnitude smaller than that of AFM. This smaller indentation of the cell’s membrane means that SICM mechanical mapping consists of mostly cytoskeletal elements, whereas AFM may include data for internal organelles, such as the nucleus. Due to this, Young’s modulus values tend to be lower when obtained by SICM compared to AFM, but trends in cellular mechanical alterations can be compared between SICM and AFM data sets [6].

A cell’s nanomechanical properties have been linked to cancer metastasis, motility and progression. It has been repeatedly reported that cancer cells show reduced Young’s modulus compared to their non-cancerous counterparts [8,9], which suggests that cell stiffness may act as a potential cancer diagnostic tool. Furthermore, changes in a cell’s cytoskeletal elements have been linked to nanomechanical alterations with enhanced F-actin bundles correlating to a higher Young’s modulus [10,11]. In contrast, the relationship between cancer metastatic ability and cellular mechanics is somewhat contested with studies relating increased metastatic potential with both an increase [12,13] and decrease in Young’s modulus [14,15]. This lack of clear understanding as to the correlation between metastatic ability and nanomechanical properties is a barrier in utilizing cell nanomechanics as a prognostic tool.

As cell stiffness could be an important overlooked biomarker for cancer, the effect of anti-cancer treatment on cellular mechanics could provide a novel insight into action, potential targets, and efficiency [16,17,18]. It has been shown that cancer cell mechanical properties may be linked to drug resistance [19], and anti-cancer drugs may alter Young’s modulus as a secondary effect [20,21]. Therefore, more investigation is needed to understand the mechanisms behind changes in cancer cell stiffness before a cell’s mechanical properties can be exploited for the benefit of patients.

Metastatic melanoma is the most aggressive form of skin cancer and is formed from the pigment-producing cells found in the basal layer of the epidermis, melanocytes. Melanoma is first formed in the epidermis where melanoma cells divide and grow horizontally (radial growth phase, RGP), followed by vertical growth into the dermis (vertical growth phase, VGP) before entering the blood or lymphatic system and spreading to a secondary site (metastatic, Met) [22]. Melanoma cell lines from the three stages of melanoma (RGP, VGP and Met) offer a useful model to study cancer development, progression and metastasis.

Thus far, most nanomechanical data acquisition has used AFM, which measures the Young’s modulus of a cell through direct contact between the cell membrane and a cantilever tip [7]. This study aims to be the first to use SICM to quantify alterations in melanoma mechanical properties across different stages. Furthermore, we aim to assess Young’s modulus changes following anti-cancer drug treatments. Paclitaxel is a potent microtubule assembly promoter, preventing effective mitosis [23]. Cisplatin activates cellular apoptotic pathways through the intrastrand cross-linking of DNA [24]. Cisplatin has also been shown to affect cell stiffness through actin accumulation in prostate cancer cells [20]. According to Cancer Research UK, dacarbazine is the most used chemotherapy drug to treat melanoma. Dacarbazine methylates DNA, causing cell apoptosis [25]. Additionally, our objective is to explore the relationship between alterations in cell cytoskeletal components, specifically F-actin and α-tubulin, and their corresponding impact on Young’s modulus.

## 2. Materials and Methods

### 2.1. Cell Lines

All melanocyte and melanoma cell lines were provided by The Functional Genomics Cell Bank, St George’s, University of London, UK. The human melanoma cell lines WM35, WM1650 and SGM3 are derived from radial growth phase melanomas. The human melanoma cell lines ME10538, WM-98-1 and WM1341B are derived from vertical growth phase melanomas. Metastatic melanoma cell lines include A375P, derived from the skin, DX3, derived from the hypodermis, and WM1158, derived from lymph nodes. A375M is a highly metastatic variant of A375P and LT5.1 is a highly metastatic variant of DX3. The human melanocyte cell line Hermes 2B was used as a non-cancerous comparison.

Melanoma cell lines were cultured in RPMI 1640 medium (Sigma-Aldrich, St. Louis, MI, USA) supplemented with final concentration 10% fetal calf serum (FCS) (Gibco, Waltham, MA, USA), 2 mM L-glutamine (Gibco), 100 units/mL penicillin (Gibco), 100 units/mL streptomycin (Gibco) and 7.5 µg/mL phenol red (Sigma-Aldrich). Cells were incubated at 37 °C and 10% CO_2_. The melanocyte cell line Hermes 2B and RGP cell line SGM3 were cultured in the previously described complete medium further supplemented with 200 nM TPA (12-O-tetradecanoylphorbol-13-acetate), 200 pM cholera toxin, 10 nM endothelin-1 and 10 ng/mL human stem cell factor. The RGP melanoma cell line WM1650 was cultured in melanoma complete culture medium further supplemented with 200 pM TPA and 200 pM cholera toxin. Differences in cell media composition between cell lines were due to the need of growth factors for melanocyte and some RGP melanoma cell lines.

### 2.2. Anti-Cancer Drugs and Reagents

Paclitaxel (Y0000698, Lot: 4.0, Sigma-Aldrich) and cisplatin (PHR1624, Lot: LRAD1147, Sigma-Aldrich) were dissolved in DMSO at a high stock concentration of 0.1 M and 0.2 M, respectively, and frozen for storage. Dacarbazine (PHR1924, Lot: LRAB1415, Sigma-Aldrich) was prepared in complete medium and warmed in a water bath until dissolved. A fresh working stock was prepared prior to each experiment. The working concentrations for drug treatments were as follows: paclitaxel 5 µM, cisplatin 1 µM and dacarbazine 20 µM. The final concentration of DMSO in the working stock of paclitaxel and cisplatin were 0.005% and 0.0005%, respectively.

### 2.3. MTT Cell Viability Assay

Cell viability following drug treatment was determined using MTT (3-(4,5-dimethylthiazol-2-yl)-2,5-diphenyltetrazolium bromide) assay (ABCAM) according to the manufacturer’s instructions. Cells were plated in a 96-well plate at 7000 cells per well and incubated for 24 h. Following incubation, the cells were incubated with varying concentrations of reagents for 24 h, including untreated and vehicle controls. Treatment media was then removed, and 50 µL serum-free RPMI media and 50 µL MTT reagent was added to each well. The plate was incubated in the dark for 3 h. The MTT reagent was then removed and 150 µL MTT solvent was added to each well. The plate was kept at room temperature on an orbital shaker in the dark for 15 min. The absorbance of the plate was read at OD = 590 nm. Each treatment was performed in triplicate and each experiment was repeated three times. Cell viability was calculated as a percentage of untreated control cells.

As paclitaxel and cisplatin stock concentrations were prepared in DMSO, a control MTT assay was used to determine at what concentration DMSO had a significant effect on cell viability. As drugs were dissolved in DMSO and stored at a high concentration, the final concentration of DMSO in the final drug treatment was maximum 0.005%. DMSO was found to have a significant effect on cell viability at 2.5% (Figure S1), and therefore changes in cell morphology could be attributed to the drug treatment due to the previously described very low DMSO final concentrations.

### 2.4. Wound Healing Assay

Cells were grown to confluence in a 12-well plate and a “wound” was made in the confluent cell layer using a 20 µL pipette tip. Cells were briefly washed in DPBS and cell media containing 1% FCS along with the treatment of choice was added. Cells were incubated for 1 h to allow floating cells to re-attach and an image was taken of the wound using a Nikon Eclipse microscope (Nikon, Tokyo, Japan). Cells were returned to the incubator and an image taken of the wound at different time intervals. The same position of the wound was imaged using a marker drawn on the base of the well. The wound healing process was analyzed using an ImageJ software plug-in and calculated on the wound closure area [26].

### 2.5. Scanning Ion Conductance Microscopy

SICM was used for the acquisition of non-contact topographic and nanomechanical maps and the experimental set up used for this study was the same as previously described by Kolomgorov et al., 2021 [6]. The SICM instrument was manufactured by ICAPPIC (ICAPPIC Ltd., London, UK) and mounted on a Nikon Eclipse Ti-2 inverted optical microscope (Nikon, Tokyo, Japan).

Nanopipettes were fabricated from borosilicate glass capillaries (O.D. 1.2 mm, I.D. 0.90 mm, 7.5 cm length) (World Precision Instruments, Sarasota, FL, USA) using a P-2000 laser puller (Shutter instruments, Novato, CA, USA). A nanopipette tip inner radius of around 50 nm was achieved using the following settings: Heat 310, Fil 3, Vel 30, Del 160, Pul 0, Heat 330, Fil 3, Vel 25, Del 160, Pul 200.

All topographic and nanomechanical scans were 50 µm by 50 µm. Image acquisition parameters were as follows: hop amplitude 2000 nm, fall rate 35 nm/ms, pre-scan sqr size 1.563 µm, pre-scan hop 3100 nm. The feedback control setpoint of 0.5% was used for topographic image acquisition and the setpoints 1 and 2% were used for nanomechanical data acquisition. As SICM cell scanning took place in an ungassed environment, cell media was changed prior to scanning to complete RPMI 1640 medium containing 20 mM final concentration HEPES solution (Sigma Aldrich).

Cells were plated at 5 × 10^4^ cells/mL in 35 mm polystyrene culture dishes. Cells were scanned up to three days after seeding. For 24 h drug treatment scans, 24 h following seeding, the required drug was added, and cells were incubated for a further 24 h.

For the 2 h treatment scans, continuous mode SICM was used. A cell was scanned twice as control images and the nanopipette raised to prevent breakage when the treatment was added. After treatment with desired final concentration, the nanopipette was lowered back onto the same cell and scanning re-commenced. As adaptive resolution was used, the time taken to complete a scan varied between cells and was usually around 10 min. To allow for easier comparison, each time point is represented as a scan number from 1–11, which represents a time period of around 2 h.

Topographic and mechanical maps were created using software created by ICAPPIC (ICAPPIC Ltd., London, UK). The average Young’s modulus of the cell was obtained using the free-hand ROI tool to select the central section of the cell and the average Young’s modulus of the area was automatically calculated by the software.

### 2.6. Immunofluorescence and Confocal Imaging

Cells were plated at 5 × 10^4^ cells/mL onto sterile glass coverslips in 4-well plates. Following incubation for 48 h, cells were treated with the desired drug and incubated for a further 24 h. Cells were fixed in 4% paraformaldehyde, permeabilized using 0.1% Triton-X and blocked with 2% donkey serum. Microtubules were stained using the primary antibody anti-alpha-tubulin mouse IgG1 monoclonal (A11126, Thermo fisher, Waltham, MA, USA) at a working concentration of 1:100. The secondary antibody, Alexa FluorTM 594 donkey anti-mouse IgG (A21203, Thermo Fisher), was used at a final concentration of 1:200. F-actin was stained using Alexa Fluor TM 488 phalloidin (A12379, Thermo Fisher) at a dilution of 1:1000. DAPI was used to stain nuclei at a concentration of 1:1000. Cells were mounted using 1:1 glycerol/PBS (Citifluor). Slides were imaged using Nikon A1R confocal microscope using 60× oil emersion lens. Images were obtained using the same image acquisition settings to allow images to be compared. Z-stack images were obtained with slices 0.75 µm apart. Corrected total cell fluorescence (CTCF) was calculated using z-stack images. A cell was manually outlined using the ImageJ freehand ROI tool and background fluorescence was measured by outlining a small area containing no cells. Corrected total cell fluorescence was calculated using the following equation:

CTCF = Integrated Density − (Area of Selected Cell × Mean Fluorescence of Background Readings)

### 2.7. Data Analysis and Statistics

All statistical data analysis was conducted using GraphPad Prism v 10.0.0. Data were tested for normality using the Shapiro–Wilk test. The statistical test used for each data set is described in the accompanying figure legend. A *p*-value less than 0.05 was considered statistically significant.

## 3. Results

### 3.1. Nanomechanical Properties of Melanoma Cell Lines with Increasing Metastatic Ability

**Figure 1:**
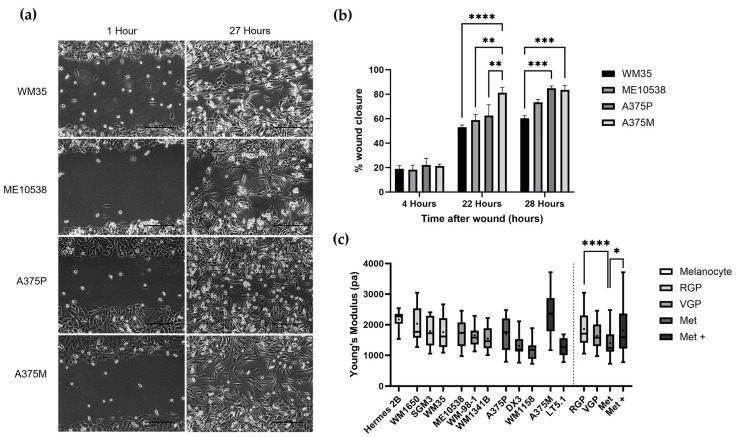
(**a**) Phase contrast scratch assay images of four melanoma cell lines from different stages (WM35—RGP, ME10538—VGP, A375P—Met, A375M—Met+). These images were analyzed using ImageJ software (v. 1.53) to calculate wound closure as a marker of metastatic ability. (**b**) Percentage wound closure over a time period of 27 h for the four melanoma cell lines. Scratch assays were conducted in duplicate in three separate experiments. Bar graph represents mean ± SEM. ** *p* < 0.01, *** *p* < 0.001, **** *p* < 0.0001; one-way ANOVA with Tukey’s multiple comparison. (**c**) The nanomechanical properties, presented as Young’s modulus (pascals), of melanoma cell lines from different stages. The dashed line separates the Young’s modulus data from individual cell lines in each stage (right hand side) and the combined average of Young’s modulus data for each stage (left-hand side). Data are presented as a box plot showing the mean (+), median, upper and lower quartiles, and minimum and maximum values. N = 15 cells for each cell line. * *p* < 0.05, **** *p* < 0.0001; Kruskal–Wallis with multiple comparisons test.

The use of SICM to measure the nanomechanical properties of single cells in physiological solution without fixation allows the exploration of cell stiffness as a potential biomarker for both cancer development and progression. Melanoma cell lines from different stages of cancer were used as a model for increasing metastatic ability with cell lines from RGP, VGP, Met and highly metastatic (Met+) stages. The commonly used scratch assay was used to validate the increasing metastatic ability of cell lines WM35, ME10538, A375P and A375M from RGP, VGP, Met and Met+ stages, respectively (Figure 1a). At 22 h of incubation, the highly metastatic cell line (A375M) saw a significantly increased wound closure compared to other cell lines, as expected (Figure 1b).

SICM was used to measure Young’s modulus (pascals) of melanoma cell lines from the different stages. A sample of SICM topography and nanomechanical maps for melanoma cell lines can be found in Figure S2. In line with previous publications that showed Young’s modulus decreases with increasing metastatic ability, we hypothesized that the Met and Met+ cell lines would show a lower Young’s modulus compared to the less metastatic melanocyte, RGP and VGP cell lines. Figure 1c shows nanomechanical data for cell lines from each of the stages of melanoma and shows a trend of decreasing Young’s modulus with increasing metastatic potential. In order to further investigate the association between nanomechanical properties and metastatic ability, Young’s modulus data from all lines from each stage were grouped. We saw a decrease in Young’s modulus average (mean ± SEM) between the RGP (1862 ± 77.80 Pa), VGP (1639 ± 60.41 Pa) and MET (1400 ± 68.02 Pa ) for all three cell lines in each stage. However, the average Young’s modulus for Met + (1814 ± 145.5 pa) was significantly higher than that of Met average. In addition, we found that the melanocyte cell line Hermes 2B had a higher Young’s modulus than VGP (*p* < 0.01, Kruskal–Wallis with multiple comparison) and Met (*p* < 0.0001, Kruskal–Wallis with multiple comparison) cell line averages, which agrees with previous studies showing that cancer cells tend to be softer than their non-cancerous counterpart. However, we saw no significant difference between melanocyte and Met+ Young’s modulus averages.

### 3.2. Nanomechanical Properties in Relation to Cytoskeletal Elements

A cell’s mechanical properties have been attributed to its cytoskeletal elements and their organization, particularly the actin cytoskeleton. In order to further understand the differences in Young’s modulus seen between the melanoma stages, cells were imaged for their F-actin and microtubule cytoskeleton. In addition, Z-stack images were analyzed for corrected total cell fluorescence (CTCF) to quantify the F-actin and α-tubulin between cell lines.

**Figure 2:**
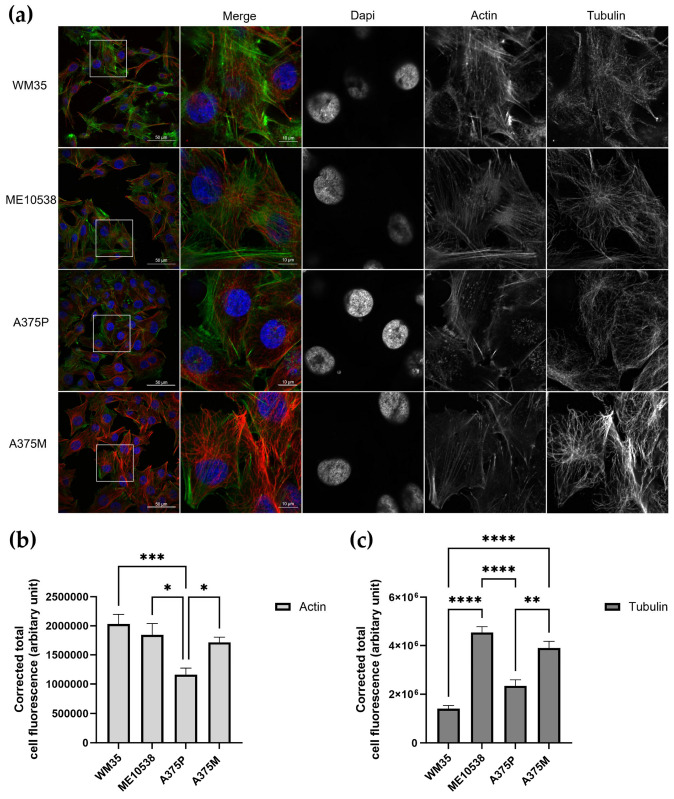
(**a**) Immunofluorescence staining of actin and tubulin cytoskeletons and confocal images for melanoma cell lines. The white box on the left-hand image corresponds to the section shown to the right. Images were taken using the same microscope image acquisition parameters. Z-stack images were obtained (slices 0.75 µm apart) and corrected total cell fluorescence values for the actin (**b**) and tubulin (**c**) cytoskeleton were calculated using ImageJ. Number of cells—WM35 *n* = 22, ME10538 *n* = 23, A375P *n* = 23, A375M *n* = 22, calculated using three Z-stack images for each cell line. * *p* < 0.05, ** *p* < 0.01, *** *p* < 0.001, **** *p* < 0.0001; Kruskal–Wallis test with multiple comparisons. Bar graphs show mean CTCF with SEM.

Immunofluorescence images of the cell lines WM35 (RGP), ME10538 (VGP), A375P (Met) and A375M (Met +) are shown in Figure 2a. We observed an F-actin CTCF pattern that closely paralleled the Young’s modulus data, revealing decreasing levels of F-actin as metastatic potential increased across the stages of RGP, VGP and Met. Furthermore, the highly metastatic cell line A375M, which saw an increased Young’s modulus, saw a significant increase in the CTCF of F-actin compared to A375P (Figure 2b). Interestingly, we saw no such pattern with the tubulin cytoskeleton, with the highest CTCF values seen in the ME10538 (RGP) and A375M (Met+) cell lines (Figure 2c).

### 3.3. Investigating Changes in Nanomechanical Properties following 24-Hour Anti-Cancer Drug Treatment

The effect of anti-cancer drugs on Young’s modulus may be an important overlooked mechanism of action. In order to investigate changes in nanomechanical properties attributed to drug treatment, A375M melanoma cells were treated for 24 h, and Young’s modulus measured using SICM. The anti-cancer drugs investigated in this study were paclitaxel, cisplatin and dacarbazine. An appropriate concentration at which to treat the A375M cells was chosen using MTT assay with IC50 values for 24 h treatment as follows: paclitaxel 20.23 µM, cisplatin 3.98 µM and dacarbazine 81.24 µM. As cytotoxicity influences cell stiffness [27], to avoid changes in cell stiffness caused by cell apoptosis, lower concentrations of 5 µM paclitaxel, 1 µM cisplatin and 20 µM were chosen.

**Figure 3:**
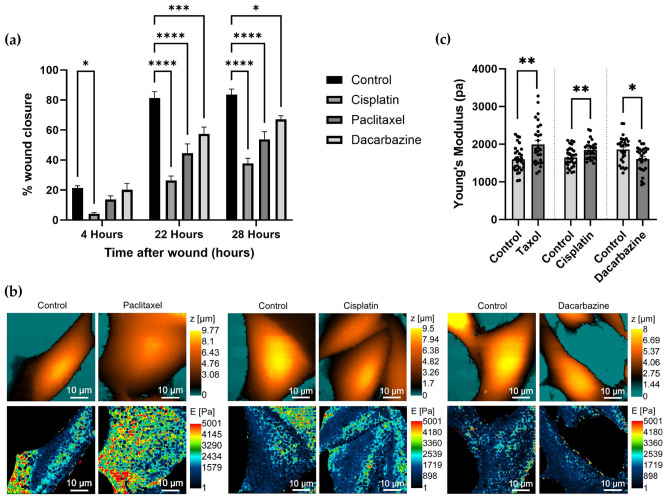
(**a**) Scratch assay wound healing for A375M cells over a time period of 27 h. Scratch assays were conducted in duplicate in three separate experiments. Bars represent mean percentage wound closure ± SEM. * *p* < 0.05, *** *p* < 0.001, **** *p* < 0.0001; one-way ANOVA with Tukey’s multiple comparison. (**b**) Example topographic (orange and blue palette) and nanomechanical (rainbow palette) scans obtained by SICM for control untreated A375M cells and A375M cells treated with anti-cancer drugs for 24 h. (**c**) Average A375M Young’s modulus measurements of control (*n* = 30) and paclitaxel-treated cells (*n* = 29). Average Young’s modulus measurements of control (*n* = 30) and cisplatin-treated cells (*n* = 28). Average Young’s modulus measurements of control (*n* = 29) and dacarbazine-treated cells (*n* = 26). * *p* < 0.05, ** *p* < 0.01; Mann–Whitney test. Bar graph represents mean Young’s modulus with SEM.

A scratch assay was used to validate the anti-migratory effects of the drugs at the concentrations previously described. Cisplatin and paclitaxel showed the highest reduction in wound closure with all drugs causing a reduction in wound closure compared to the untreated control (Figure 3a). Once an anti-migratory effect on the A375M cells had been established, 24 h drug pre-treated cells were scanned using SICM to obtain topography and cell nanomechanical images (Figure 3b). Cells did not see adverse changes in topography after treatment. The effect of each treatment on cell stiffness varied between treatments with paclitaxel and cisplatin treatment causing a statistically significant increase in Young’s modulus. In contrast, dacarbazine treatment saw a decrease in Young’s modulus compared to the untreated control (Figure 3c).

**Figure 4:**
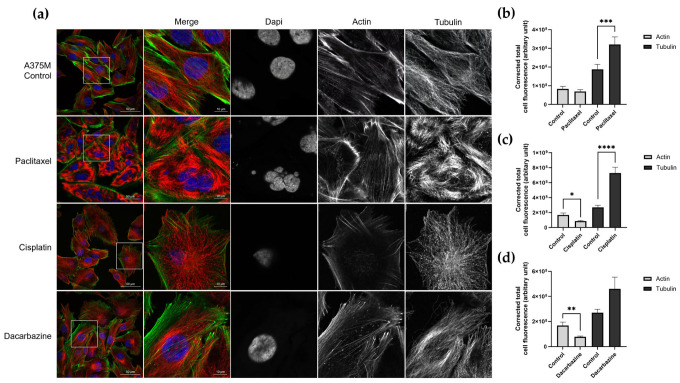
(**a**) Immunofluorescence staining of F-actin and α-tubulin cytoskeletons and confocal images of A375M cells after 24 h treatment with anti-cancer drugs. The white box on the left-hand image corresponds to the section shown to the right. Images were taken using the same microscope parameters. Z-stack images were obtained (0.75 µm slices) and corrected total cell fluorescence for the actin and tubulin were calculated using ImageJ. (**b**) The average CTCF with SEM is shown for paclitaxel (control *n* = 56, treated *n* = 35), (**c**) cisplatin (control *n* = 30, treated *n* = 25), (**d**) dacarbazine (control *n* = 30, treated *n* = 29). * *p* < 0.05, ** *p* < 0.01, *** *p* < 0.001, **** *p* < 0.0001; Mann–Whitney test. Bar graph shows mean with SEM.

As previously discussed, the cytoskeleton plays an important role in nanomechanics, and changes in cytoskeletal organization brought about by drug treatment may cause changes in cellular nanomechanics. Following Young’s modulus data collection after 24 h drug treatment, we sought to understand the changes in the F-actin and α-tubulin cytoskeleton, which may be the cause of the Young’s modulus increase or decrease. Immunofluorescence confocal microscopy images for untreated control A375M cells and treated cells can be found in Figure 4a. As expected, following the Young’s modulus data, paclitaxel caused the most apparent changes in the α-tubulin cytoskeletal organization. Paclitaxel promotes microtubule assembly preventing efficient mitosis, which can clearly be seen by the α-tubulin immunofluorescence staining with microtubule bundles and multinucleated nuclei. The CTCF saw a statistically significant increase in tubulin, as expected (Figure 4b). Cisplatin treatment saw an increase in Young’s modulus, and α-tubulin was found to be significantly increased despite a reduction in the CTCF of F-actin (Figure 4c). Similarly, CTCF analysis for dacarbazine treatment saw a significant decrease in F-actin but a non-significant increase in tubulin and thus a Young’s modulus decrease was observed (Figure 4d).

### 3.4. Changes in Nanomechanical Properties following 2-Hour Anti-Cancer Drug Treatment

As SICM is a non-contact method of nanomechanical data acquisition, as well as measuring Young’s modulus changes before and after the treatment of different cell populations, it also allows for continuous scanning to measure Young’s modulus changes in the first two hours after treatment. This offers a novel investigation as it allows Young’s modulus of the same cell to be tracked over time both before and after treatment. Furthermore, the non-contact, high resolution data acquisition of SICM ensures that cells are not damaged during scanning, as seen with AFM.

**Figure 5:**
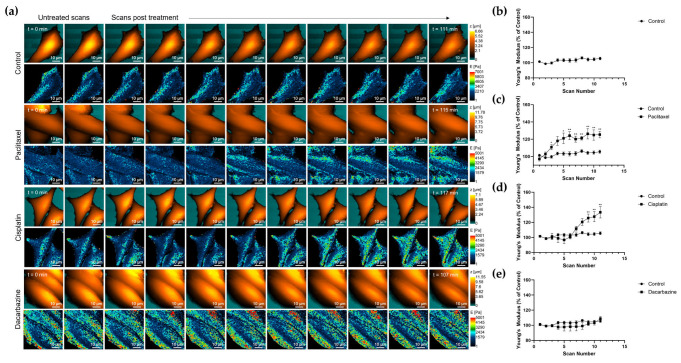
(**a**) Topography and corresponding nanomechanical images obtained using continuous scanning mode SICM. Two scans were taken of an A375M cell, the treatment was added to the cell media and scanning continued. (**b**) The mean Young’s modulus of a cell was plotted with increasing scan numbers to show changes in cell stiffness over time (approximately 2 h). The control cells (*n* = 5) had fresh medium added following the second scan. The following treatments were compared to the control Young’s modulus plot and can be found in each of the average plots for (**c**) paclitaxel (*n* = 5), (**d**) cisplatin (*n* = 6) and (**e**) dacarbazine (*n* = 5). * *p* < 0.05, ** *p* < 0.01; Mann–Whitney test. Plots show mean Young’s modulus of all cells with SEM.

A375M cells were scanned twice prior to treatment to obtain an untreated control Young’s modulus measurement. The treatment was then added to the cell media and scanning continued following the same cell for nine further scans (approximately 1.5 h after treatment). Continuous scans for each treatment can be found in Figure 5a, and changes in Young’s modulus over time can be seen. Control cells saw a slight increase in Young’s modulus on average, but dynamic changes in Young’s modulus are expected as cells are alive and unfixed (Figure 5b). Paclitaxel treatment showed an increase in Young’s modulus, with the average Young’s modulus becoming statistically significantly higher than the control average from scan five (Figure 5c). This is in line with the effect seen after 24 h treatment. Cisplatin treatment for 2 h also saw a statistically significant increase above the control average Young’s modulus from scan nine (Figure 5d). Finally, dacarbazine treatment saw no significant change in Young’s modulus compared to the control average with heterogeneity in the effect on cell stiffness among cells. (Figure 5e). Nanomechanical plots over time for individual cells can be found in Figure S3.

## 4. Discussion

In this study, we have demonstrated the use of SICM in measuring the Young’s modulus of individual, unfixed melanoma cells. Our investigation aimed to establish the connection between a cell’s nanomechanical properties and metastatic ability, as well as explore the impact of anti-cancer drugs on Young’s modulus both over 24 h and 2 h periods. Cancer biomarkers are important tools in diagnosis and prognosis. However, there is a lack of universal, accurate and reliable markers to distinguish between cancer and non-cancer cells as well as cancer cells at different stages. Cellular nanomechanics has been suggested as a biomarker as there have been studies to show that cancer cells have a lower Young’s modulus than their non-cancerous counterparts [28,29]. Indeed, we found the Young’s modulus of non-cancerous melanocytes was significantly higher than that of cells from the VGP and MET stages of melanoma. The link between Young’s modulus and metastatic ability, particularly in melanoma, is somewhat debatable with publications showing both an increase and decrease in Young’s modulus with increasing metastatic ability [13,15,30,31].

This study marks the first instance of utilizing SICM to explore the relationship between melanoma nanomechanics and metastatic capability, in contrast to prior inquiries that have relied on AFM. Due to the difference in cell indentation between AFM and SICM, the Young’s modulus values obtained may not be directly compared. As the indentation value of the method contributes to the cell stiffness measurement and heterogeneity between cell lines, the comparison of AFM Young’s modulus values may also vary between studies [32]. As with other comparative studies, the trends in Young’s modulus between cell lines or treatments may be compared between AFM and SICM data [6].

Our findings reveal a pattern of decreasing Young’s modulus in melanoma cell lines across stages with increasing metastatic ability, although there is some variability between cells within and between cell lines of the same stage. This aligns with the observations made by Watanabe et al., 2012, where they reported an inverse relationship between nanomechanical stiffness obtained with AFM and migratory ability [15]. Conversely, we found that the Young’s modulus of the highly metastatic cell line A375M was increased even above that of the RGP cell lines. This is contradictory to the belief that metastatic cells have a lower Young’s modulus to aid in motility and spread in the body [9]. A similar trend was observed by Weder et al., 2014, with the metastatic melanoma cell line WM239A having a higher Young’s modulus when measured with AFM than RGP and VGP cell lines. Weder et al. proposed that this increased cell stiffness may be due to increased plasticity of metastatic cells. Indeed, it has been shown that the extracellular matrix plays a crucial role in metastasis, and metastatic cells may exhibit a higher level of adaptability in order to survive and proliferate at a secondary site in the body [13,33]. Our findings suggest that nanomechanics may not be a universal and conserved marker of cancer metastatic ability, and therefore its usefulness as a diagnostic and prognostic tool requires careful reevaluation and thorough investigation. However, a cell’s nanomechanical properties may still offer insight into cancer progression and may be a useful tool when used alongside other markers such as contractility and certainly warrants further investigation [34].

Understanding the effect of anti-cancer drug treatment on a cell’s nanomechanical properties may give insight into unknown modes of action, secondary mechanisms to reduce cancer spread and drug effectiveness. Most previous studies investigating changes in Young’s modulus using AFM following drug treatment rely upon measuring cell stiffness 24 h post treatment. As well as examining alterations in Young’s modulus following 24 h anti-cancer drug treatment, this study employed SICM to offer a fresh perspective on the impact of anti-cancer drugs on Young’s modulus during the initial two hours of treatment using continuous scan mode. Twenty-four-hour paclitaxel treatment saw an increase in A375M Young’s modulus. This was to be expected due to the microtubule stabilizing mode of action of the drug. These data are in line with previous studies using AFM to show that paclitaxel and docetaxel (a microtubule stabilizing agent in the same taxane group of chemotherapy drugs) increases Young’s modulus [20,35,36]. Despite no known cytoskeletal target, cisplatin has recently been attributed to causing a higher cell stiffness through actin accumulation [20]. Here, we also saw an increase in Young’s modulus following 24 h cisplatin treatment. Due to no known cytoskeletal target, there is a lack of research showing nanomechanical changes following dacarbazine treatment. To our knowledge, only one other study has measured Young’s modulus after dacarbazine treatment, in which the authors found that dacarbazine decreased Young’s modulus with lower fluorescence intensity of actin and tubulin cytoskeletons [37]. Similarly, we saw a reduction in the Young’s modulus of cells treated with dacarbazine over a 24 h period. As the 24 h treatment data were comparable to that of previous studies which employed AFM, we sought to understand nanomechanical changes in the first 2 h of treatment using continuous scan mode which utilizes the non-contact method of SICM to continuously scan the same cell before and after treatment without causing cell damage. We found that both paclitaxel and cisplatin increased Young’s modulus during the first 1.5 h of treatment whilst dacarbazine saw no significant change in Young’s modulus during this time. Our data show significant changes in cell stiffness upon treatment with some anti-cancer drugs when compared to an untreated control. Despite the very low final treatment concentration of DMSO and no effect on cell viability seen at these concentrations, further investigation is needed to ensure that there is no effect of DMSO on cell stiffness. We wish to also highlight that these data are the first to investigate such changes and that these data offer validation of the use of SICM continuous scan mode to investigate nanomechanical changes following short term drug treatment. There is a need for further investigation before drawing significant conclusions, and this offers an exciting future perspective on nanomechanical data acquisition.

The relationship between a cell’s nanomechanical properties and the cytoskeleton has been widely published. In particular, the actin cytoskeleton and stress fibers have been found to contribute to a cell’s mechanical properties [38,39], and high tubulin expression has been linked to lower melanoma survival [40]. Here, we examined if changes in Young’s modulus were attributed to F-actin and microtubule organization or levels via corrected total cell fluorescence. The CTCF of F-actin between melanoma cells from different stages mirrored the Young’s modulus data, with the stiffer cell lines showing higher actin levels alongside thicker actin bundles, which aligns with previous studies [5,41]. However, tubulin levels varied between cell lines with the highest level seen by RGP and Met+ cell lines, which suggests that the tubulin cytoskeleton may play a lesser role in a cell’s mechanical properties. Despite this, treatment with paclitaxel saw a reduction in CTCF F-actin and a large increase in α-tubulin, with a severe change in the microtubule organization in the cell. Similarly, cisplatin treatment saw a significant decrease in F-actin and a significant increase in α-tubulin, contrary to previous publications, which saw increased Young’s modulus due to increased actin stress fibers [42]. A reduction in Young’s modulus following 24 h dacarbazine treatment can be linked to a reduction in F-actin as previously reported [37]. Our data demonstrate that perhaps both cytoskeletal organization and expression play a role in maintaining a cell’s mechanical properties. Furthermore, there are other factors which may affect Young’s modulus, such as myosin II [43] and P-cadherin [44], which warrants further investigation to fully understand cancer cell nanomechanics and its correlation with metastasis and treatment.

## 5. Conclusions

This study offers a new insight into the usefulness of a cell’s nanomechanical properties as a cancer diagnostic and prognostic tool as well as its use to investigate anti-cancer drug mechanisms and effectiveness. According to our results, both the actin and tubulin cytoskeleton networks play an integral role in modulating Young’s modulus and these networks may be modified by anti-cancer drugs to affect cell motility, mitosis and viability. However, this work shows the complicated and contradictory field of cancer cellular mechanics and highlights the variation between studies. The continuous scan mode of non-contact SICM Young’s modulus measurement offers an independent investigation into an important and often overlooked potential biomarker for cancer.

## Data Availability

The data used in the publication are not hosted on any resources and can be provided privately upon request.

## Supplementary Materials

The following supporting information can be downloaded at: https://www.mdpi.com/article/10.3390/cells12192401/s1, Figure S1: MTT data for DMSO cell viability; Figure S2: A sample of SICM topographic and cell stiffness maps for melanoma cell lines; Figure S3: SICM stiffness plots for the five individual A375M cells scanned following anti-cancer drug treatment.
