# Supplementary material for: Measuring Melanoma Nanomechanical Properties in Relation to Metastatic Ability and Anti-Cancer Drug Treatment Using Scanning Ion Conductance Microscopy
Source: Cells. 2023 Oct 4;12(19):2401. doi: 10.3390/cells12192401 (PMC10571876; doi:10.3390/cells12192401)
Supplement: Supplementary file 1 [file cells-12-02401-s001.zip › cells-2614440-supplementary.pdf]

Measuring Melanoma Nanomechanical Properties in Relation to Metastatic Ability and Anti-Cancer Drug Treatment Using Scanning Ion Conductance Microscopy

Supplementary information

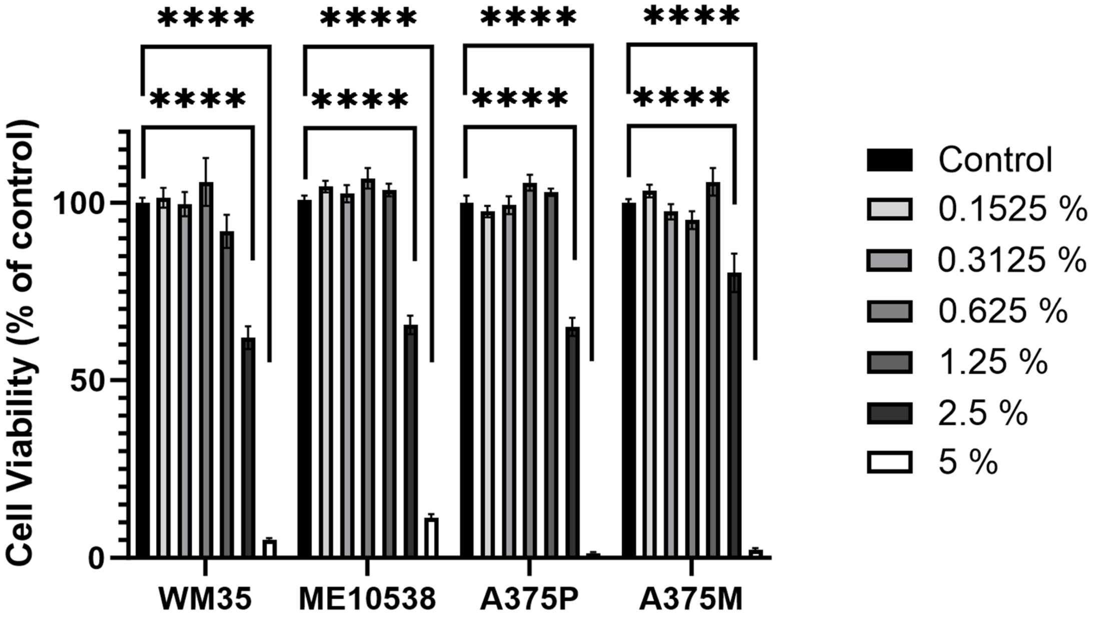

Figure S1: Cell viability assay at different concentrations of DMSO for four melanoma cell lines. DMSO was added to complete culture medium at the concentrations shown with a control cell treatment containing no DMSO. Cell viability was assessed using an MTT assay. Assays were conducted in triplicate and bars show the mean ± SEM for three experiments. **** $P < 0.0001$, One-way ANOVA.

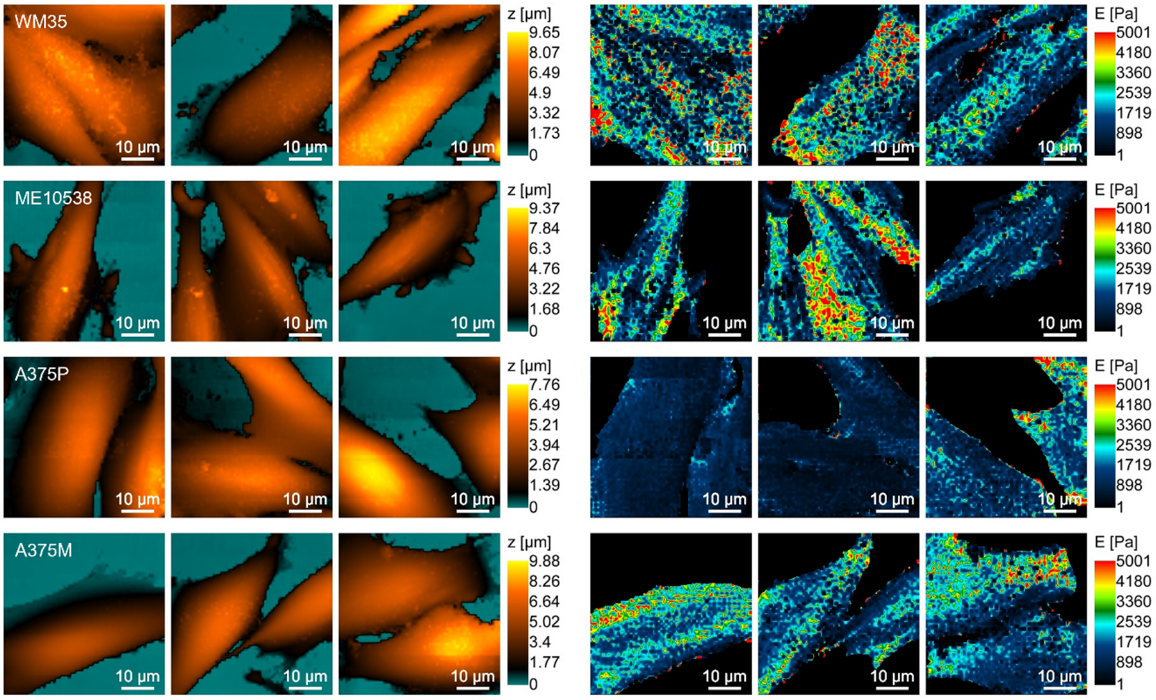

Figure S2: A sample of SICM topographic and cell stiffness maps for melanoma cell lines. Topographic images (shown in blue and orange palette) can be found on the left panel and the corresponding stiffness maps (rainbow palette) can be found on the right panel. These scans are from four melanoma cell lines WM35, ME10538, A375P and A375M which are from the RGP, VGP, Met and Met+ stages respectively.

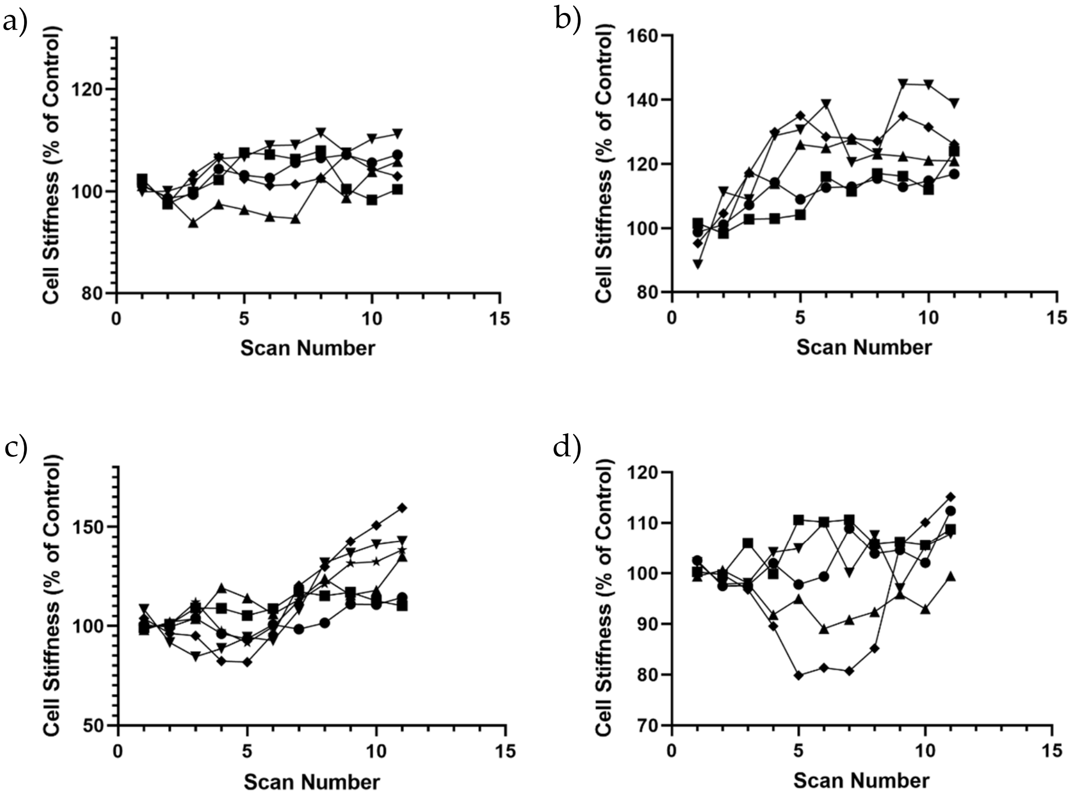

Figure S3: SICM stiffness plots for the five individual A375M cells scanned following anti-cancer drug treatment. The total scan time is around 2 hours. Cell stiffness is represented as a percentage of control (the first two untreated scans). **(a)** A plot of cell stiffness change over time for untreated control cells. **(b)** A plot of cell stiffness change over time for cells treated with paclitaxel. **(c)** A plot of cell stiffness change for cells treated with cisplatin. **(d)** A plot of cell stiffness change for cells treated with dacarbazine. Each point represents the mean stiffness of the cell at that time point. Each connected line on the plot represents a single cell. Control, paclitaxel and dacarbazine n = 5 cells, cisplatin n = 6 cells.
